# Phase-Specific Changes in Vital Signs and Electrocardiogram Findings During Hyperbaric Oxygen Therapy in Hemodynamically Stable Patients: A Prospective Observational Study

**DOI:** 10.3390/jcm15051725

**Published:** 2026-02-25

**Authors:** Seon Tae Kim, Jeong Mi Lee, Jeong Woo Choi

**Affiliations:** 1 Department of Emergency Medicine, Wonkwang University School of Medicine, Iksan 54538, Republic of Korea; kst5478@naver.com; 2 Department of Public Health, Graduate School of Wonkwang University, Iksan 54538, Republic of Korea

**Keywords:** hyperbaric oxygen therapy, blood pressure, heart rate, electrocardiogram, oxygen toxicity, vital signs, oxygen saturation

## Abstract

**Background/Objectives**: Physiological changes during hyperbaric oxygen therapy (HBOT) are not well characterized, particularly in non-emergent patients receiving HBOT as part of a repeated or maintenance treatment course, in whom understanding physiological responses during individual sessions is important for clinical monitoring. This study evaluated changes in vital signs and electrocardiographic (ECG) findings across the pre-compression, compression, maintenance, decompression, and post-treatment phases and evaluated clinical symptoms. **Methods**: This prospective observational study enrolled 50 hemodynamically stable non-emergent patients undergoing HBOT at a single tertiary center. Changes in vital signs and ECG findings were recorded across all phases. Repeated vital sign measurements were analyzed using linear mixed models; ECG abnormalities were assessed using generalized linear mixed models. **Results**: Heart rate decreased significantly across all HBOT phases compared with baseline. Blood pressure (BP) remained stable during compression and maintenance but increased significantly during decompression and post-treatment. Respiratory rate decreased during treatment and then returned to baseline. Oxygen saturation remained within normal ranges throughout all phases. Transient ECG rhythm abnormalities were observed in 10.0% of patients, primarily during compression and maintenance phases. One patient developed brief clinical symptoms accompanied by supraventricular tachycardia immediately after decompression, which resolved spontaneously without intervention. No significant oxygen toxicity or serious adverse events were observed. **Conclusions**: HBOT in hemodynamically stable non-emergent patients induces predictable, largely transient physiological changes and is well tolerated under standard protocols. Blood pressure elevation was most pronounced during decompression and the post-treatment phase, whereas transient ECG abnormalities were observed primarily during the compression and maintenance phases, with a single episode of supraventricular tachycardia occurring immediately after decompression. These findings provide foundational clinical data for understanding phase-specific physiological responses during HBOT and inform future studies in higher-risk patient populations.

## 1. Introduction

Hyperbaric oxygen therapy (HBOT) is a noninvasive therapeutic modality in which patients breathe 100% oxygen for approximately 90–120 min in a specially designed chamber pressurized at 2–3 atmospheres absolute [1]. Hyperbaric oxygen chambers are classified as monoplace chambers, which accommodate a single patient, or multiplace chambers, which allow for the simultaneous treatment of multiple patients. Although treatment pressure and duration may vary depending on institutional equipment and clinical indications, HBOT typically results in a marked increase in arterial oxygen partial pressure, thereby improving tissue hypoxia and various physiological effects that underlie its therapeutic benefits [2,3].

HBOT is used for emergent conditions such as carbon monoxide intoxication, decompression sickness, air embolism, and gas gangrene, as well as for non-emergent indications, including diabetic foot ulcers (DFUs), burns, compromised skin grafts or flaps, radiation injury, refractory osteomyelitis, and sudden sensorineural hearing loss (SSNHL) [4].

HBOT is a relatively safe treatment modality, with a low reported incidence of complications [5]. Adverse events associated with HBOT are broadly categorized into pressure-related injuries (barotrauma) and oxygen toxicity. Middle ear barotrauma is the most common complication and may manifest as ear pain, hemotympanum, hearing impairment, or tympanic membrane rupture [6,7]. Oxygen toxicity occurs less frequently; however, it may affect the central nervous, respiratory, and cardiovascular systems and is characterized by symptoms such as dizziness, chest pain, syncope, shortness of breath, and seizures [8]. These manifestations may overlap with clinical signs of hemodynamic instability, such as arrhythmias, hypotension, or decreased oxygen saturation, potentially leading to diagnostic uncertainty when these overlapping presentations are interpreted without sufficient monitoring data.

Continuous real-time monitoring of vital signs and consciousness level during HBOT is essential in patients with emergent conditions or severe illness who exhibit hemodynamic instability (e.g., unstable blood pressure [BP], heart rate [HR], oxygen saturation, or altered mental status). Because symptoms during HBOT may overlap between expected physiological responses and adverse events (including oxygen toxicity), careful clinical monitoring and interpretation are essential, particularly in emergent or high-risk populations. In contrast, patients undergoing HBOT for non-emergent indications are typically hemodynamically stable and often receive repeated maintenance therapy. Therefore, HBOT in these patients is frequently administered without additional monitoring devices or supplementary testing in the absence of overt symptoms or signs. Although HBOT is typically administered as a course of repeated sessions, the present study focused on phase-specific physiological responses during a single representative session in clinically stable patients, to characterize short-term safety and monitoring considerations rather than cumulative treatment effects. Consequently, systematic clinical data describing phase-specific changes in vital signs and electrocardiographic (ECG) findings during HBOT are limited, and evidence linking physiological changes to oxygen toxicity-related symptoms during treatment remains insufficient. This lack of data represents a significant limitation in assessing the safety of HBOT in routine clinical practice.

HBOT may induce cardiovascular effects such as peripheral vasoconstriction, increased blood pressure, bradycardia, and reduced cardiac output. However, most of these findings are derived from comparisons of vital signs at single time points before and after treatment or from retrospective analyses. Therefore, prospective studies that systematically assess physiological changes across each treatment phase are limited [9,10,11]. Furthermore, some studies have examined physiological responses to hyperbaric environments in healthy adults or divers; however, investigations systematically evaluating physiological changes during HBOT in clinical patient populations remain scarce [12,13]. In particular, few Korean studies have comprehensively analyzed hemodynamic parameters, such as BP, HR, respiratory rate (RR), and oxygen saturation (SpO_2_), or ECG changes during HBOT in patients treated for approved clinical indications. Moreover, clinical studies assessing the relationship between oxygen toxicity-related symptoms and concurrent changes in vital signs or ECG findings during HBOT are exceedingly rare.

Therefore, this study was designed based on the hypothesis that HBOT may induce measurable changes in vital signs and ECG findings during treatment and potentially result in clinical symptoms, even in patients who are hemodynamically stable before treatment. To test this hypothesis, this study continuously assessed changes in vital signs and ECG findings across five predefined phases, which included pre-compression, compression, maintenance, decompression, and post-treatment, and evaluated the occurrence of clinical symptoms. The primary objective of the study was to provide descriptive clinical reference data on phase-specific physiological responses and symptom occurrence during HBOT in non-emergent patient populations, thereby informing routine safety monitoring and supporting clinical interpretation when symptoms arise.

## 2. Materials and Methods

### 2.1. Study Design

This prospective observational study was designed to evaluate changes in vital signs and ECG findings during HBOT and to analyze associated clinical symptoms. The study was conducted at a single tertiary care center, Wonkwang University Hospital (Iksan, Republic of Korea), and included 50 inpatients and outpatients undergoing HBOT who had provided written informed consent. This study was conducted and reported in accordance with the Strengthening the Reporting of Observational Studies in Epidemiology (STROBE) guidelines.

Eligible participants were patients receiving HBOT for non-emergent indications, including SSNHL, DFUs, Buerger disease, radiation-induced tissue necrosis, burns, compromised skin grafts or flaps, postoperative wounds after digital replantation, and refractory osteomyelitis. Patients were excluded if they had contraindications to HBOT, such as untreated pneumothorax, active middle ear disease, or severe claustrophobia. Only patients with at least one prior HBOT session and the ability to equalize pressure during compression and decompression using maneuvers such as the Valsalva maneuver were included to minimize the effects of pressure-related injury. Additional exclusion criteria were hemodynamic instability before treatment (systolic BP [SBP] < 90 mmHg, HR < 60 beats/min, SpO_2_ < 95%, or RR ≥ 25 breaths/min), uncontrolled hypertension (SBP ≥ 160 mmHg or diastolic BP [DBP] ≥ 100 mmHg), impaired consciousness or inability to communicate symptoms clearly, and missing baseline clinical or physiological data required for analysis. These exclusion criteria were applied to ensure hemodynamic stability and patient safety during non-emergent HBOT and to facilitate characterization of physiological responses within a clinically stable population. Patients with baseline bradycardia were excluded to minimize confounding from pre-existing autonomic or conduction abnormalities and to focus on physiological changes attributable to HBOT itself.

Sample size was calculated using G*Power software (version 3.1.9.7; Heinrich-Heine-Universität Düsseldorf, Düsseldorf, Germany). To detect significant changes in HR before and after HBOT, an effect size of 0.4, a two-sided α level of 0.05, and a power of 0.80 were applied based on prior studies [14]. The minimum required sample size was calculated as 45 patients. The target enrollment was set at 50 patients, allowing for an anticipated dropout rate of approximately 10.0%. Of the 52 patients initially enrolled, two withdrew for personal reasons; therefore, 50 patients were included in the final analysis.

**Figure 1:**
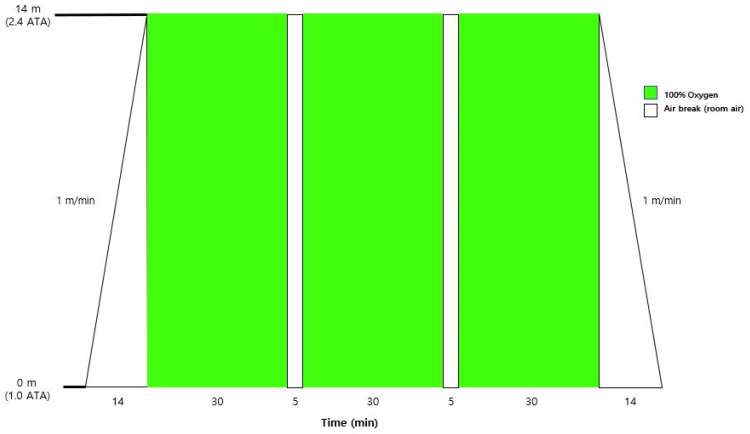
Institutional hyperbaric oxygen therapy (HBOT) protocol used in this study. Patients were treated in a multiplace hyperbaric chamber according to a protocol based on the US Navy Treatment Table 9 hyperbaric oxygen treatment protocol with minor institutional modifications. Compression and decompression were performed at a rate of 1 m/min to a target pressure equivalent to 14 m seawater (2.4 atmospheres absolute [ATA]). The protocol consisted of three 30-min periods of 100% oxygen breathing separated by 5-min air breaks. Green blocks indicate 100% oxygen breathing, whereas white blocks indicate air breaks.

HBOT was administered using a medical-grade hyperbaric chamber (HBOT-DL600-2400; Interocean Co., Ltd., Busan, Republic of Korea) installed at Wonkwang University Hospital. Treatment pressure and duration followed the institutional clinical protocol based on the indications recommended by the Undersea and Hyperbaric Medical Society [3]. The protocol was derived from the U.S. Navy Treatment Table 9 (USN TT9) hyperbaric oxygen treatment protocol, commonly used in multiplace chambers, with selected parameters (e.g., compression rate) adjusted according to institutional safety and operational policies (Figure 1).

Vital signs and ECG data were obtained using a patient monitoring system approved by the Ministry of Food and Drug Safety (BPM-770; BIONICS, Hongcheon, Republic of Korea). The system enabled stable physiological signal acquisition under hyperbaric conditions and was used for continuous monitoring during HBOT. The same monitoring device was used throughout the entire treatment session for all patients.

This study was conducted in accordance with the ethical principles of the Declaration of Helsinki [15] and was approved by the Institutional Review Board of Wonkwang University Hospital (approval no. WKUH 2022-01-016). Written informed consent was obtained from all participants. Access to personal information and study data was restricted to investigators who had completed institutional ethics training.

### 2.2. Data Collection

**Figure 2:**
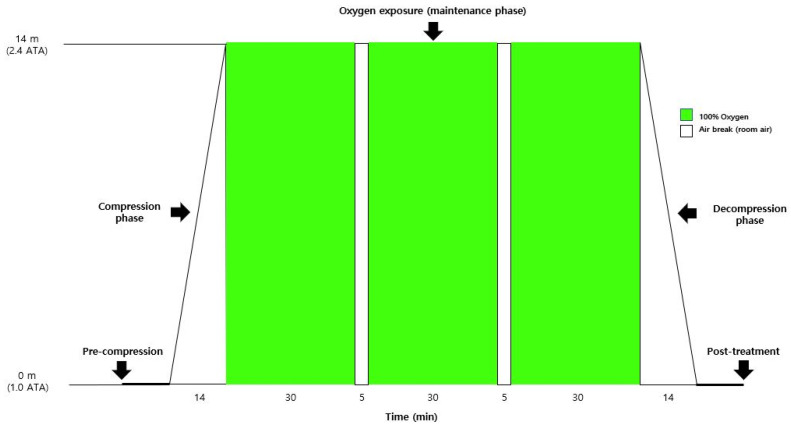
Schematic diagram of the HBOT protocol and predefined monitoring phases. Changes in vital signs and electrocardiography were continuously monitored and analyzed at five predefined phases: pre-compression (10 min before the start of compression at 1.0 ATA), compression phase (5–10 min after the initiation of pressurization), oxygen exposure (maintenance phase) (during the second oxygen breathing period at 2.4 ATA), decompression phase (5–10 min after the initiation of decompression), and post-treatment (10 min after completion of decompression). Green blocks indicate periods of 100% oxygen breathing, whereas white blocks indicate air breaks.

Vital signs and ECG findings were continuously recorded across five predefined HBOT phases: pre-compression (10 min before compression initiation), compression (5–10 min after compression initiation), maintenance (during the second oxygen-breathing period), decompression (5–10 min after initiation of decompression), and post-treatment (10 min after completion of decompression) (Figure 2). Recorded variables included SBP, DBP, HR, RR, SpO_2_, and ECG findings. For the compression and decompression phases, physiological measurements were obtained during the mid-phase interval (5–10 min after initiation) to represent stable responses during pressure change, while avoiding transient early effects and variability near phase completion.

ECG monitoring was performed continuously using a three-lead configuration from the pre-compression phase through the post-treatment phase to enable rhythm surveillance. Initial recognition of ECG changes was performed by trained emergency medical technicians working as hyperbaric chamber operators, and all suspected findings were subsequently reviewed and classified by the attending physician. For the purpose of statistical analysis, an ECG abnormality was defined as any newly detected rhythm or conduction finding deviating from normal sinus rhythm, excluding changes attributable to external artifacts such as patient movement or electrode interference. Recorded ECG changes included premature atrial or ventricular contractions, supraventricular or ventricular tachyarrhythmias, atrioventricular conduction disturbances, and other rhythm changes observed during continuous monitoring. Twelve-lead ECG was not routinely obtained as part of the study protocol, and detailed assessment of myocardial ischemia, repolarization abnormalities, or precise arrhythmia subtyping was beyond the scope of this monitoring approach. Although ECG monitoring was continuous throughout the HBOT session, formal ECG data used for phase-based analyses were recorded only at these predefined phase-specific time points.

RR could not be measured automatically by the monitoring system and was therefore measured manually over a 60-s period while patients were at rest and not speaking, with pauses in breathing included in the count. Measurements were performed by two trained hyperbaric chamber operators following a standardized protocol, typically by a single operator at each phase, at a single representative time point during each phase rather than averaged across the interval. Observers were aware of the treatment phase, as measurements were conducted in real time during chamber operation.

Clinical symptoms were collected throughout the entire treatment session using a structured case report form based on patient-reported complaints and observed findings. Recorded symptoms included chest discomfort, palpitations, shortness of breath, dizziness, syncope, and seizures.

Demographic and clinical characteristics collected for each participant included age, sex, height, weight, body mass index (BMI), comorbidities (hypertension, diabetes mellitus, cardiovascular disease, pulmonary disease, and other conditions), current medications, primary diagnosis, and HBOT session number at the time of study participation. Cardiovascular disease was defined as a history of arrhythmia, heart failure, or prior myocardial infarction. Hypertension was recorded separately as a baseline comorbidity; patients with uncontrolled hypertension were excluded, whereas individuals with well-controlled hypertension receiving stable medical therapy were included.

### 2.3. Statistical Analysis

Continuous variables are presented as the mean ± standard deviation. To evaluate temporal changes in SBP, DBP, HR, RR, and SpO_2_ measured during HBOT, linear mixed-effects models were applied for continuous variables (SBP, DBP, HR, RR, and SpO_2_). The treatment phase was included as a fixed effect, and participant-specific random intercepts were included to account for within-participant correlation arising from repeated measurements. Models were adjusted for sex, age, diabetes mellitus, cardiovascular disease, other comorbidities, and body mass index (BMI). ECG abnormalities (binary variable) were analyzed using generalized linear mixed-effects models. Covariates could not be included owing to model variance estimation limitations; therefore, the treatment phase was included as the fixed effect, and a random intercept for each participant was used to account for within-participant correlation. Model assumptions were evaluated by visual inspection of residual plots to assess normality and homoscedasticity, and no major violations were identified. Missing data were minimal and were handled within the mixed-effects modelling framework without imputation. Model parameters for linear mixed-effects models were estimated using restricted maximum likelihood, whereas generalized linear mixed-effects models were estimated using maximum likelihood. For continuous variables, pre-compression values were used as the reference, and changes during compression, maintenance, decompression, and post-treatment were presented as regression coefficients (β) with 95% confidence intervals. For binary variables, results were presented as odds ratios (ORs) with 95% confidence intervals. All statistical analyses were performed using IBM SPSS Statistics version 29.0 (IBM Corp., Armonk, NY, USA). A two-sided *p*-value < 0.05 was considered statistically significant.

### 2.4. Use of Generative Artificial Intelligence (AI)

Generative AI (ChatGPT-4o, OpenAI, San Francisco, CA, USA) was used in a limited capacity to assist with English language editing and refinement of sentence structure during manuscript preparation. The AI tool was used only to improve clarity and readability of the text and was not involved in the study design, data analysis, interpretation of results, or generation of scientific conclusions. All content was critically reviewed and approved by the authors, who take full responsibility for the integrity and accuracy of the manuscript.

## 3. Results

### 3.1. Participant Characteristics

Detailed baseline demographic characteristics, clinical diagnoses, comorbidities, and baseline vital signs are summarized in Table 1. A total of 50 patients were included in the final analysis. The study population consisted predominantly of middle-aged adults, with a higher proportion of male participants. All patients were hemodynamically stable at the initiation of HBOT. Sudden sensorineural hearing loss was the most common indication for HBOT, followed by compromised skin grafts or flaps, radiation injury, burns, and DFU.

Diabetes mellitus was the most frequently observed comorbidity, whereas a small proportion of patients had underlying cardiovascular disease, including arrhythmia and a history of myocardial infarction. One patient with atrial fibrillation was present at baseline. The patient remained hemodynamically stable throughout the treatment; therefore, they were included in the analysis.

### 3.2. Changes in Vital Signs by HBOT Phases

Linear mixed-effects model analysis using the pre-compression phase as the reference demonstrated phase-dependent changes in vital signs after adjusting for sex, age, diabetes mellitus, cardiovascular disease, other comorbidities, and BMI (Table 2). Because the mixed-effects models were adjusted for covariates, the intercept values represent adjusted estimates at reference covariate levels and should not be interpreted as raw baseline means, which are presented separately in Table 1.

**Table 2:** Linear mixed-effects model analysis of changes in vital signs across HBOT phases.

Oxygen Therapy Phases	β	SE	95% CI	*p*-Value
Systolic blood pressure				
Intercept (pre-compression)	95.8	20.7	53.9–137.6	<0.001
Compression phase	−2.4	1.6	−5.6–0.8	0.136
Maintenance phase	1.7	1.6	−1.5–4.9	0.290
Decompression phase	9.1	1.6	6–12.3	<0.001
Post-treatment	6.0	1.6	2.8–9.1	<0.001
Diastolic blood pressure				
Intercept (pre-compression)	61.0	15.0	30.7–91.2	<0.001
Compression phase	−0.6	1.1	−2.9–1.6	0.588
Maintenance phase	1.2	1.1	−1.1–3.5	0.296
Decompression phase	5.1	1.1	2.9–7.4	<0.001
Post-treatment	5.3	1.1	3.1–7.6	<0.001
Respiratory rate				
Intercept (pre-compression)	17.3	2.7	11.8–22.9	<0.001
Compression phase	−1.0	0.3	−1.7 to −0.3	0.004
Maintenance phase	−2.0	0.3	−2.7 to −1.3	<0.001
Decompression phase	−1.7	0.3	−2.4 to −1	<0.001
Post-treatment	−0.5	0.3	−1.2–0.2	0.146
Heart rate				
Intercept (pre-compression)	82.2	12.6	56.7–107.7	<0.001
Compression phase	−3.2	0.8	−4.8 to −1.6	<0.001
Maintenance phase	−10.4	0.8	−12 to −8.9	<0.001
Decompression phase	−11.2	0.8	−12.8 to −9.7	<0.001
Post-treatment	−7.4	0.8	−9 to −5.8	<0.001
Oxygen saturation				
Intercept (pre-compression)	98.9	0.5	97.9–100	<0.001
Compression phase	1.0	0.1	0.7–1.2	<0.001
Maintenance phase	1.0	0.1	0.8–1.3	<0.001
Decompression phase	1.2	0.1	0.9–1.4	<0.001
Post-treatment	0.9	0.1	0.6–1.2	<0.001

Notes: Values are presented as β coefficients with corresponding 95% confidence intervals derived from linear mixed-effects models. The intercept represents the adjusted estimated mean at the reference levels of covariates included in the model and therefore does not correspond to the unadjusted baseline or phase-specific means. Observed (unadjusted) descriptive baseline values are presented in Table 1, while adjusted phase-specific trends are illustrated in Figure 3. Models were adjusted for sex, age, body mass index, diabetes mellitus, heart disease, and other diseases. β, regression coefficient; SE, standard error; CI, confidence interval.

**Figure 3:**
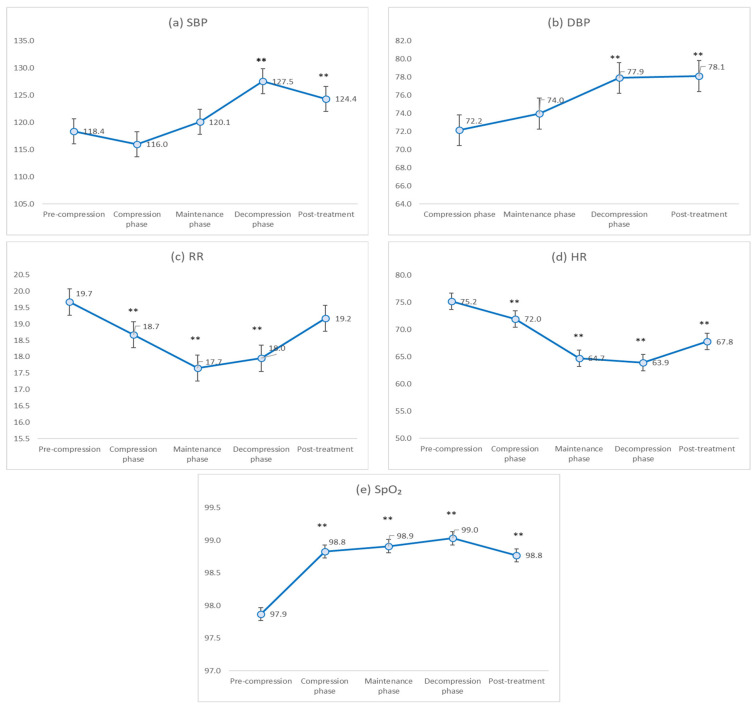
Adjusted estimated means of vital signs across HBOT phases derived from linear mixed-effects models. Values are adjusted for sex, age, body mass index, diabetes mellitus, heart disease, and other comorbidities. (**a**) Systolic blood pressure (SBP), (**b**) diastolic blood pressure (DBP), (**c**) respiratory rate (RR), (**d**) heart rate (HR), and (**e**) oxygen saturation (SpO_2_). Adjusted means with 95% confidence intervals are shown. ** *p* < 0.01 compared with baseline (pre-compression).

SBP did not differ significantly from baseline during compression (β = −2.4 mmHg, *p* = 0.136) or maintenance (β = 1.7 mmHg, *p* = 0.290). In contrast, SBP increased significantly during decompression (β = 9.1 mmHg, *p* < 0.001) and remained significantly elevated during post-treatment (β = 6.0 mmHg, *p* < 0.001) (Figure 3a).

Compared with baseline, DBP showed no significant change during compression (β = −0.6 mmHg, *p* = 0.588) or maintenance (β = 1.2 mmHg, *p* = 0.296). However, a significant increase was observed during decompression (β = 5.1 mmHg, *p* < 0.001), which persisted during post-treatment (β = 5.3 mmHg, *p* < 0.001) (Figure 3b).

RR decreased significantly during compression (β = −1.0 breaths/min, *p* = 0.004), maintenance (β = −2.0 breaths/min, *p* < 0.001), and decompression (β = −1.7 breaths/min, *p* < 0.001). RR remained lower than baseline during post-treatment; however, the difference was not significant (β = −0.5 breaths/min, *p* = 0.146) (Figure 3c).

HR decreased significantly compared with baseline across all phases. The mean reduction was 3.2 beats/min during compression (β = −3.2, *p* < 0.001), with a greater decrease during maintenance (β = −10.4, *p* < 0.001). A significant reduction persisted during decompression (β = −11.2, *p* < 0.001) and post-treatment (β = −7.4, *p* < 0.001) (Figure 3d).

SpO_2_ increased significantly compared with baseline during compression (β = 1.0%, *p* < 0.001), maintenance (β = 1.0%, *p* < 0.001), decompression (β = 1.2%, *p* < 0.001), and post-treatment (β = 0.9%, *p* < 0.001). No clinically meaningful desaturation or respiratory compromise was observed throughout treatment (Figure 3e).

Although all participants met hemodynamic stability criteria at baseline, during decompression, 7 patients (14.0%), and during the post-treatment phase, 6 patients (12.0%), transiently exceeded predefined blood pressure thresholds (SBP ≥160 mmHg and/or DBP ≥100 mmHg). These transient elevations were more frequently observed in patients with pre-existing cardiovascular comorbidities or hypertension and resolved without the need for clinical intervention.

In addition to the β estimates derived from the linear mixed-effects models, phase-specific adjusted mean values are presented in Figure 3 to facilitate clinical interpretation of the absolute magnitude of physiological changes across HBOT phases.

### 3.3. ECG Findings by HBOT Phases

All 50 patients underwent continuous three-lead ECG monitoring throughout HBOT, and any rhythm abnormalities other than normal sinus rhythm were documented by phase. ECG abnormalities were infrequent across all HBOT phases, with only a small number of events observed across the entire session. In generalized linear mixed-effects models, no statistically significant differences were observed in the occurrence of ECG abnormalities during compression (OR = 0.23, *p* = 0.07), maintenance (OR = 0.23, *p* = 0.07), decompression (OR = 1.00, *p* = 1.00), or post-treatment (OR = 1.00, *p* = 1.00) compared with the pre-compression phase (Table 3). However, odds ratio estimates, particularly during decompression and post-treatment, were associated with wide confidence intervals, reflecting limited statistical power due to the low number of events. Therefore, these findings should be interpreted descriptively rather than as evidence of equivalence between phases.

One patient (2.0%) demonstrated persistent atrial fibrillation from baseline through the entire treatment course, without developing additional arrhythmias. Excluding this patient, ECG changes were observed in five patients (10.0%) during HBOT (Table 4). All observed changes were newly detected during treatment and were transient. One patient exhibited recurrent premature ventricular contractions (PVCs) during both compression and maintenance. The remaining four patients demonstrated single, phase-limited events: PVCs during compression in two patients, PVCs during maintenance in one patient, and atrioventricular block (AVB) during maintenance in one patient.

By phase, ECG changes occurred in three patients (6.0%) during compression (all PVCs) and in three patients (6.0%) during maintenance (two PVCs and one AVB). No new ECG abnormalities were observed during decompression or post-treatment within the predefined phase definitions.

All ECG changes were transient, confined to specific phases, and resolved without persistence after treatment completion. No patient developed sustained arrhythmia or required clinical intervention. One additional case of ECG change associated with clinical symptoms occurred immediately after decompression but outside the predefined phases and is described separately in the clinical symptom analysis.

### 3.4. Clinical Symptoms During Treatment

Clinical symptoms during HBOT were observed in one patient (2.0%), who reported transient chest discomfort and palpitations immediately after completion of decompression while still inside the chamber, with symptoms lasting approximately 2 min. Concurrent three-lead ECG monitoring demonstrated supraventricular tachycardia (SVT). Both symptoms and ECG findings resolved spontaneously without medical intervention.

Because the arrhythmia was transient, resolved spontaneously, and was not associated with hemodynamic instability, twelve-lead ECG was not obtained. This event occurred immediately after decompression and before the predefined post-treatment phase (10 min after completion of decompression); therefore, it was excluded from the phase-based ECG analysis and is reported descriptively as an immediate post-decompression clinical event. No other patients experienced clinically significant symptoms during HBOT.

## 4. Discussion

Reduced HR was the most consistent and prominent finding across all treatment phases, consistent with previously reported decreased HR during exposure to hyperbaric and hyperoxic conditions [12,16,17], and in line with more recent clinical reviews describing hyperoxia-induced increases in systemic vascular resistance and vagally mediated bradycardic responses during HBOT [18]. Additionally, a similar, consistent reduction in HR was observed across all phases compared with baseline. This response is frequently attributed to peripheral vasoconstriction with a subsequent increase in arterial pressure, leading to baroreceptor stimulation and baroreflex activation, with the resulting autonomic modulation favoring increased parasympathetic tone and reflex bradycardia [17]. Studies in healthy adults have similarly demonstrated reductions in HR and alterations in HR variability during hyperbaric and hyperoxic exposure, with increased high-frequency components reflecting parasympathetic predominance [12], suggesting that hyperoxic exposure may affect cardiovascular autonomic regulation. The present findings suggest that these autonomic mechanisms are present in clinical patient populations undergoing HBOT.

In contrast to HR, BP showed phase-dependent changes. No significant differences were observed during compression or maintenance compared with baseline; however, SBP and DBP increased significantly during decompression and remained elevated in the post-treatment phase. Prior studies evaluating BP responses to HBOT have reported heterogeneous findings, including recent observational data demonstrating variable BP elevations depending on underlying cardiovascular comorbidities [10,19]. For example, in patients with diabetes mellitus or hypertension, repeated HBOT is associated with increased SBP and DBP, with more pronounced effects observed in patients with underlying cardiovascular disease [9]. Conversely, other studies have reported minimal BP changes in patients without hypertension but significant post-treatment BP elevation in patients with a history of hypertension, with differences in BP changes and timing of monitoring across studies [10].

By administering 100% oxygen at pressures exceeding atmospheric levels, HBOT enhances tissue oxygenation by increasing the amount of oxygen dissolved in plasma [20]. However, exposure to hyperbaric and hyperoxic conditions can also induce peripheral vasoconstriction and increase systemic vascular resistance, which may manifest as hemodynamic responses such as elevated BP [17]. This hyperoxic vasoconstriction has been attributed to the increased production of reactive oxygen species during hyperoxic exposure and a concomitant reduction in endothelial nitric oxide bioavailability [21]. In this study, BP elevation was primarily observed during decompression and post-treatment. This finding suggests that changes in BP may not be solely driven by hyperoxic exposure itself but by a combination of factors occurring during treatment termination, including changes in autonomic regulation, patient movement, relief of psychological tension, and transient sympathetic activation or dynamic changes in vascular tone as oxygen partial pressure rapidly declines during decompression. Such phase-specific patterns are challenging to capture in studies relying solely on single pre- and post-treatment measurements, and the current phase-based analytical approach provides a more granular assessment of physiological responses during HBOT.

RR decreased significantly during compression, maintenance, and decompression and returned to baseline levels after treatment completion. This reduction in RR may be related to physiological suppression of ventilatory drive under hyperoxic conditions owing to altered chemoreceptor responsiveness, a well-described mechanism [22]. Throughout all treatment phases, SpO_2_ remained within the normal range with a modest increase, and no clinically significant hypoxemia or respiratory failure was observed. Given the hyperbaric hyperoxic conditions of HBOT, this increase in SpO_2_ reflects an expected physiological response and should be interpreted cautiously because of ceiling effects inherent to pulse oximetry; nevertheless, the observed reduction in respiratory rate was not accompanied by clinically adverse respiratory outcomes in these hemodynamically stable patients.

Clinical symptoms occurring during HBOT may arise from multiple etiologies, including oxygen toxicity, physiological hemodynamic responses to treatment, or cardiovascular events such as arrhythmias, necessitating careful clinical differentiation. Complications during HBOT are broadly categorized as pressure-related injury and oxygen toxicity. Oxygen toxicity may affect the central nervous, cardiovascular, and respiratory systems [8]. Clinically, the incidence of oxygen toxicity-related seizures is low, and most cases are transient with limited associations with long-term neurologic sequelae [20]. Central nervous system oxygen toxicity is a rare complication of HBOT and is generally described as a time- and pressure-dependent phenomenon associated with sustained hyperoxic exposure and characteristic neurological manifestations, including seizures or altered mental status [23]. Such events are most commonly reported during periods of sustained oxygen exposure rather than after termination of hyperoxia [3,23]. However, in this study, findings suggestive of central nervous system oxygen toxicity, such as seizures or altered level of consciousness, were not observed within the predefined treatment phases. The findings of this study are consistent with prior literature and support the conclusion that HBOT can be administered with a high level of safety when performed under standard clinical protocols [8,12,16,17,20].

Only one patient reported transient chest discomfort and palpitations lasting approximately 2 min immediately after decompression, accompanied by a temporary ECG finding consistent with SVT. The event occurred outside the predefined treatment phases, and both symptoms and ECG changes resolved spontaneously without additional medical intervention. The SVT episode observed in this study differs from the characteristic clinical presentation of oxygen toxicity when considering the brief duration, spontaneous resolution, absence of altered consciousness or neurologic symptoms, and immediate post-decompression timing. HBOT may elicit cardiovascular responses through coronary vasoconstriction, myocardial oxygen supply–demand imbalance, and alterations in autonomic regulation, which may contribute to the development of transient arrhythmias [2,24]. Accordingly, the observed episode is more appropriately interpreted as a cardiovascular response occurring in the context of physiological changes during HBOT rather than being attributed solely to oxygen toxicity. These findings highlight the importance of a comprehensive clinical evaluation when symptoms occur during HBOT, while considering cardiovascular events as well as oxygen toxicity.

As this study was conducted in hemodynamically stable non-emergent patients, the observed physiological changes were generally mild and transient. However, the finding that HBOT may induce peripheral vasoconstriction, changes in BP, and a reduced HR may have greater clinical relevance in patients with underlying cardiovascular disease or in critically ill populations. Systematic reviews have reported circulatory adverse effects associated with HBOT, including chest pain, arrhythmias, and pulmonary edema, highlighting the need for careful patient selection and vigilant monitoring during treatment in higher-risk patient groups [5,8].

This study has some limitations. Because respiratory rate was measured manually by observers who were aware of the treatment phase, some degree of observer-related variability and reactivity bias cannot be excluded. Sex was recorded as a biological variable, and baseline characteristics were reported separately for males and females; however, this study was not designed or powered to evaluate sex-specific differences in physiological responses to HBOT. Future studies with larger cohorts are warranted to determine whether sex-related differences influence phase-specific responses during HBOT.

Second, the study population was heterogeneous with respect to clinical indications for HBOT, including sudden sensorineural hearing loss, chronic wounds, burns, and graft-related conditions. Although all participants were hemodynamically stable and treated according to a standardized HBOT protocol, the limited sample size precluded meaningful subgroup analyses according to indication or comorbidity. Therefore, the present findings should be interpreted as reflecting overall physiological responses to HBOT in stable patients, rather than indication-specific effects, and caution is warranted when extrapolating these results to higher-risk populations.

Additionally, the analytical scope of this study was limited to a single HBOT session. Although only one symptomatic event was observed during the analyzed session, additional review outside the study protocol revealed that the same patient experienced three additional symptoms over the course of the full treatment period. Because these events occurred beyond the predefined observation window, they were not included in the quantitative analysis. Moreover, physiological variables were assessed at predefined representative time points during each treatment phase rather than continuously averaged, and therefore peak transient responses during compression or decompression may not have been fully captured. Consequently, findings derived from a single-session analysis are insufficient to fully evaluate cumulative risk or the potential for oxygen toxicity associated with repeated exposure.

Furthermore, ECG monitoring in this study was limited to a three-lead configuration, which restricts the detection of repolarization changes, ischemic patterns, and detailed arrhythmia characterization. Accordingly, ECG findings should be interpreted as reflecting rhythm changes during HBOT rather than comprehensive assessments of cardiac electrophysiology or ischemic risk. In addition, the low incidence of ECG abnormalities in this cohort further limited statistical power, increasing the risk of a type II error in detecting rare arrhythmic events.

Beyond these ECG-related methodological limitations, the exclusion of patients with baseline bradycardia (heart rate < 60 bpm) or reduced oxygen saturation (SpO_2_ < 95%) may have introduced selection bias by excluding individuals at potentially higher cardiopulmonary risk. As a result, the observed heart rate reduction and respiratory stability during HBOT may not fully represent responses in patients with baseline bradycardia, hypoxemia, or greater physiological vulnerability. Similarly, although hypertension was present in a subset of patients, individuals with uncontrolled hypertension were excluded; therefore, the potential influence of hypertension on blood pressure responses during HBOT may have been attenuated in this cohort. Furthermore, because some participants had well-controlled hypertension at baseline, subtle effects of antihypertensive treatment on blood pressure responses during HBOT cannot be entirely excluded. Detailed information on specific antihypertensive medications, such as beta-blockers or calcium channel blockers, was not systematically available in this outpatient cohort; therefore, their potential modulatory effects on heart rate and blood pressure responses during HBOT could not be assessed.

Consistent with this limitation, the risk of oxygen toxicity is influenced by a single exposure as well as by repeated treatments, cumulative oxygen dose, and individual susceptibility [25]. Accordingly, the present results should be interpreted as an assessment of physiological responses and short-term safety within the context of a single treatment session. Future studies should evaluate cumulative effects across repeated HBOT sessions. Furthermore, this study was a single-center prospective observational study restricted to hemodynamically stable non-emergent patients. The absence of an external control group is one limitation, which was addressed by applying a self-controlled design in which each participant served as their own reference using pre-treatment values. To account for repeated measurements, analytical validity was further addressed through the use of linear and generalized linear mixed-effects models. Additionally, only three-lead ECG monitoring was performed, limiting a more detailed characterization of arrhythmic events. Thus, these findings have limited generalizability to emergent settings or to patients with significant underlying cardiovascular disease. Furthermore, cumulative effects and long-term physiological changes associated with repeated treatments could not be assessed, as only a single HBOT session was analyzed. Therefore, multicenter and long-term follow-up studies should be conducted to address these limitations.

Nonetheless, this study is clinically meaningful in that it prospectively characterized phase-specific physiological changes during HBOT in a real-world patient population. By addressing an important gap in the existing literature, these findings provide foundational data to inform future investigations involving higher-risk populations and extended treatment protocols.

## 5. Conclusions

During HBOT, HR decreased consistently across treatment phases, whereas BP remained stable during compression and maintenance but increased significantly during decompression and the post-treatment phase. RR decreased transiently during treatment and returned to baseline after treatment completion. Overall, no clinically harmful physiological changes were observed, and the responses identified in this study were largely mild and transient, supporting the relative safety of HBOT when performed under standard clinical protocols in hemodynamically stable non-emergent patients.

Because participants were clinically stable and had completed at least one prior HBOT session, these findings most directly apply to non-emergent patients adapted to routine maintenance HBOT and may not generalize to first-session or higher-risk populations. Although these physiological changes were not clinically significant in this stable cohort, the possibility that similar responses may have greater clinical implications in patients with underlying cardiovascular disease or hemodynamic instability cannot be excluded. By providing prospective, phase-specific physiological data from a clinical patient cohort, this study offers foundational evidence to inform structured safety monitoring during HBOT and to support future investigations evaluating cumulative physiological responses across repeated treatment sessions.

## Figures and Tables

**Table 1:** Baseline demographic and clinical characteristics of participants before hyperbaric oxygen therapy (HBOT).

Variables	*n* (%) or Mean ± SD
Age (years), mean ± SD	53.2 ± 15.5
Sex, *n* (%)	
Male	33 (66.0)
Female	17 (34.0)
Body mass index (kg/m^2^), mean ± SD	24.5 ± 3.8
Primary diagnosis, *n* (%)	
SSNHL	31 (62.0)
Burns	4 (8.0)
DFU	1 (2.0)
Post-graft	9 (18.0)
Radiation	5 (10.0)
Comorbidities, *n* (%)	
Diabetes mellitus	13 (26.0)
Cardiovascular disease *	4 (8.0)
Hypertension	6 (12.0)
Other diseases †	15 (30.0)
Systolic blood pressure (mmHg), mean ± SD	121.9 ± 15.9
Diastolic blood pressure (mmHg), mean ± SD	77.6 ± 12.1
Heart rate (beats/min), mean ± SD	75.9 ± 12.7
Respiratory rate (breaths/min), mean ± SD	19.2 ± 2.7
Oxygen saturation (%), mean ± SD	97.8 ± 1.2
Atrial fibrillation, *n* (%)	1 (2.0)

* Cardiovascular disease included arrhythmia, heart failure, or prior myocardial infarction. † Other diseases included malignancy and other non-cardiovascular chronic conditions; categories are not mutually exclusive. DFU, diabetic foot ulcer; SD, standard deviation; SSNHL, sudden sensorineural hearing loss.

**Table 3:** Association between treatment phase and ECG abnormalities using a generalized linear mixed model.

	OR	95% CI	*p*-Value
Intercept (pre-compression)	Reference		
Compression phase	0.23	0.05–1.13	0.070
Maintenance phase	0.23	0.05–1.13	0.070
Decompression phase	1.00	0.18–5.55	1.000
Post-treatment	1.00	0.18–5.55	1.000

Notes: ECG abnormalities were defined as any newly detected rhythm or conduction findings deviating from normal sinus rhythm, as specified in the Methods. Because of the low number of ECG events, odds ratio estimates are associated with wide confidence intervals and are intended for descriptive interpretation rather than inferential comparison. CI, confidence interval; OR, odds ratio.

**Table 4:** Comparison of ECG status between baseline and each treatment phase.

		Compression Phase		Maintenance Phase		Decompression Phase		Post-Treatment	
		−	+	−	+	−	+	−	+
ECG change	−	46 (92.0)	3 (6.0)	46 (92.0)	3 (6.0)	49 (98.0)	0 (0.0)	49 (98.0)	0 (0.0)
	+	0 (0.0)	1 (2.0)	0 (0.0)	1 (2.0)	0 (0.0)	1 (2.0)	0 (0.0)	1 (2.0)
	Total	46 (92.0)	4 (8.0)	46 (92.0)	4 (8.0)	49 (98.0)	1 (2.0)	49 (98.0)	1 (2.0)

Notes: Table 4 summarizes the presence (+) or absence (−) of ECG changes detected within each predefined phase window. ECG changes were defined as any newly detected rhythm or conduction findings deviating from normal sinus rhythm, as specified in the Methods. The post-treatment phase was defined as the 10-min period after completion of decompression. One episode of supraventricular tachycardia occurred immediately after decompression and before the predefined post-treatment phase and is therefore reported separately in the Results. ECG, electrocardiographic.

## Data Availability

The data presented in this study are available upon reasonable request from the corresponding author. De-identified individual-level data can be provided under a data-sharing agreement, subject to institutional and ethical approval. Where appropriate, a data dictionary and summary outputs from the statistical analyses can also be shared to facilitate reproducibility.

## Abbreviations

The following abbreviations are used in this manuscript:

AVB	Atrioventricular block
BP	Blood pressure
CI	Confidence interval
DBP	Diastolic blood pressure
DFU	Diabetic foot ulcer
HBOT	Hyperbaric oxygen therapy
ECG	Electrocardiographic
HR	Heart rate
OR	Odds ratio
PVC	Premature ventricular contraction
RR	Respiratory rate
SBP	Systolic blood pressure
SSNHL	Sudden sensorineural hearing loss
SVT	Supraventricular tachycardia
